# The renal damage and mechanisms relevant to antitumoral drugs

**DOI:** 10.3389/fonc.2023.1331671

**Published:** 2023-12-12

**Authors:** Jiyu Tang, Nan Yang, Shujun Pan, Peiyao Ren, Maosheng Chen, Juan Jin, Qiang He, Yuqun Zeng

**Affiliations:** ^1^ The Second School of Clinical Medicine, Zhejiang Chinese Medical University, Hangzhou, China; ^2^ Department of Nephrology, Zhejiang Provincial People’s Hospital (Affiliated People’s Hospital), Hangzhou Medical College, Hangzhou, China; ^3^ Jinzhou Medical University, Graduate School of Clinical Medicine, Jinzhou, China; ^4^ School of Medicine, Hangzhou Normal University, Hangzhou, Zhejiang, China; ^5^ School of Chinese Medicine, Faculty of Medicine, The Chinese University of Hong Kong, Hong Kong, Hong Kong SAR, China; ^6^ Department of Nephrology, The First Affiliated Hospital of Zhejiang Chinese Medical University (Zhejiang Provincial Hospital of Traditional Chinese Medicine), Hangzhou, China

**Keywords:** antineoplastic drugs, nephrotoxicity, renal damage, mechanisms, early markers

## Abstract

Over the past few decades, significant progress has been made in the development of drugs to combat cancer. It is unfortunate that these drugs can also lead to various kidney injuries and imbalances in electrolyte levels. Nephrotoxicity caused by chemotherapy drugs can impact different parts of the kidneys, including the glomeruli, renal tubules, interstitium, or renal microvessels. Despite the existing knowledge, our understanding of the mechanisms underlying the renal damage caused by antitumoral drugs remains incomplete. In this review, we aim to provide a comprehensive overview of the specific types of kidney injury and the mechanisms responsible for the drug-mediated renal damage, and briefly discuss possible prevention and treatment measures. Sensitive blood and urine biomarkers can provide clinicians with more information about kidney injury detection and reference value for subsequent treatment options. In addition, we emphasize that both oncologists and nephrologists have a responsibility to remain vigilant against the potential nephrotoxicity of the drugs. It’s crucial for experts in both fields to collaborate in early detection, monitoring and prevention of kidney damage.

## Introduction

Antitumor drugs are mainly divided into alkylating agents, antitumor antibiotics, antitumor hormones, metal platinum, anti-metabolites, antitumor plants, and molecular targeted drugs, etc. Among them, alkylating agents are simple in synthesis with an important value for tumor treatment, while antibiotic drugs exert a good therapeutic effect on malignant tumors, but with the poor selectivity, extensive adverse reactions and easy development of drug resistance. As new anticancer drugs specifically targeting cancer cells are emerging, the prognosis of cancer patients is slowly improving, but the targeting and toxicity of drugs are two major problems. The kidneys are an important component of xenobiotic metabolism, and nephrotoxicity remains an unavoidable complication that may lead to adjustment or discontinuation of an anticancer therapy, thus affecting patient survival. Many drug-related nephrotoxicities do not have a clear mechanism of injury or pathophysiology, which poses a specific challenge to clinicians in selecting their treatment strategies. For instance, traditional chemotherapy drugs, such as cisplatin and alkylating agent ifosfamide, etc., often act on renal tubules, resulting in acute tubular necrosis or injury. In contrast, molecularly targeted drugs may cause glomerular lesions characterized by podocyte disease or thrombotic microangiopathy. This article provides a comprehensive review of the occurrence and underlying mechanisms of renal function insufficiency associated with the commonly used drugs. A key focus is on reducing the frequency and severity of nephrotoxicity resulting from chemotherapy drugs. To achieve this goal, researchers should emphasize the evaluation of early biomarkers for kidney injury, especially ones obtainable through noninvasive techniques. By employing such noninvasive methods, clinicians can promptly detect kidney damage and intervene accordingly. This proactive approach will contribute to minimizing the incidence and severity of drug-induced nephrotoxicity, ultimately improving patient outcomes.

## Platinum-derived compounds

### Mechanisms and clinical manifestations

**Figure 1:**
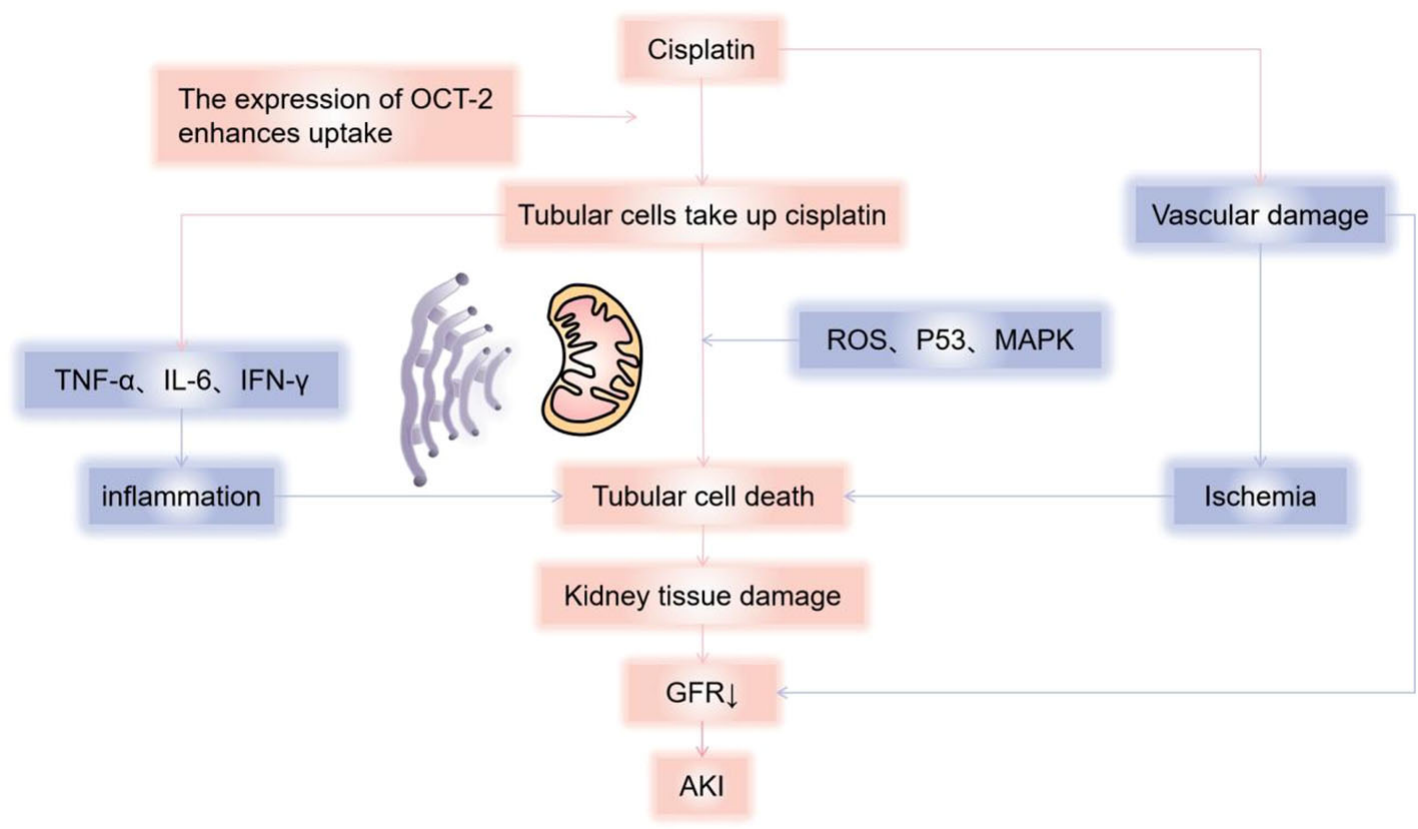
Mechanisms of cisplatin causing kidney damage. Cisplatin causes renal dysfunction through a variety of mechanisms, including toxicity to tubular epithelial cells, vasoconstriction, and pro-inflammatory effects of renal microvessels. Cisplatin enters cells through organic cation transporters (OCTs), which activates signaling pathways (p21) that promote cell death (MAPK, p53, ROS, etc.) or cell protection, and damage the renal tubules, mainly reflected in apoptosis and necrosis of the S3 segment of the proximal tubule, distal curved tubule and collecting duct, resulting in a decrease in glomerular filtration rate. According to current reports, it is mainly mitochondria-triggered intrinsic pathways, extrinsic pathways mediated by TNF (tumor necrosis factor) receptor/ligand, and endoplasmic reticulum stress pathways. Cisplatin can also cause renal vascular damage, resulting in ischemic tubular cell death and decreased glomerular filtration rate (GFR). AKI, Acute kidney injury; GFR, Glomerular Filtration Rate.

Cisplatin is approved for the treatment of many types of cancers (1) and is the most nephrotoxic chemotherapy drug detected early. The most common and important clinical manifestation of cisplatin nephrotoxicity is acute kidney injury(AKI). In the first 24 hours after cisplatin administration, with more than 50% of the drug excreted in the urine, and the concentration of platinum reached in the renal cortex is several times higher than in plasma and other organs. Drugs that accumulate in the urinary system cause renal dysfunction through a variety of mechanisms, including toxicity to tubular epithelial cells, vasoconstriction and proinflammatory effects of renal microvessels (2) (**Figure 1**). The toxicity to renal tubular cells is mainly reflected in apoptosis and necrosis of the S3 segment of the proximal tubule, distal curved tubule and collecting duct. The expression of proximal tubular organic cation transporter-2 (OCT2) enhances renal uptake, mediating the accumulation of cisplatin in proximal tubular epithelial cells (3). In addition, renal microvasoconstriction is observed after cisplatin injection, resulting in reduced renal blood flow. The renal response to cisplatin also includes the increased expression of proinflammatory cytokines (4, 5). Cisplatin has the ability to decrease the expression and activity of glucose and amino acid transporters that rely on sodium, as well as magnesium and water transporters. Furthermore, it hinders the metabolism of glutathione and cysteine-glycine conjugates, and also suppresses the production of reactive oxygen species. These mechanisms help to explain the manifestation of Fanconi-like syndrome often observed in patients undergoing cisplatin therapy. When cisplatin is used in combination with bleomycin or gemcitabine, thrombotic microangiopathy(TMA) may lead to direct endothelial injury and secondary platelet activation (6–9). Renal impairment is dose-dependent, and we have summarized the incidence of AKI in several studies reported in **Table 1** (6, 42, 43). In a recent study of 821 adult cancer patients with multiple tumor types treated with cisplatin, after 6 years of follow-up, 31.5% patients developed AKI, with an estimated initial reduction in median eGFR of 10mL/min/1.73m^2^. Most patients have a small but permanent decrease in eGFR without progression to end-stage renal disease requiring dialysis (10). Other renal manifestations include hypomagnesemia, hypokalemia, sodium depletion, Fanconi-like syndrome, and anemia (**Table 2**) (7, 44, 52).

**Table 1:** The incidence of nephrotoxicity caused by anticancer drugs.

Authors, year	Types of study	Sample size	Tumor types	Medications	Nephrotoxicity	Incidence rate
Sheron Latcha et al. 2016 (10)	Rretrospective study	821	Diverse tumor types	Cisplatin	AKI	31.5%
Francesco T revisani et al. 2022 (11)	Retrospective, observational cohort study	292	NSCLC	Cisplatin (35%), Carboplatin(25%)	AKI	Cisplatin 10.9%
						Carboplatin 6.8%
Jori May et al. 2014 (12)	Retrospective Analysis	194	Lymphoma (72.8%), sarcoma (21%)	High dose methotrexate	AKI	Lymphoma 9.1%
						Sarcoma 1.5%
Tracy Wiczer et al. 2016 (13)	Retrospective, single-center review	140	Leukemia or lymphoma	High dose methotrexate	AKI	38.6%
Sheron Latcha et al. 2022 (14)	Large adult cohort study	865	A variety of malignancies	High dose methotrexate	AKI	Overall group 32.1%
						Survivorship group 37.4%
Omar Abdel-Rahman et al. 2014 (15)	Meta-review	5694	Solid tumors	Ramucirumab	Proteinuria	All grade proteinuria 5.8 to 17%
D. Arnold et al. 2017 (16)	Meta-review	4996	Gastric/gastroesophageal junction, NSCLS and metastatic colorectal cancers	Ramucirumab	Proteinuria	3.1%-9.4%
Ze-Feng Zhang et al. 2014 (17)	Meta -review	798	Solid tumors	Pazopanib	Proteinuria	All grade 13.5%
						High grade 2.2%
Michael J Sorich et al. 2016 (18)	Pooled secondary analysis	1392	mRCC	Pazopanib or Sunitinib	Any grade (1–4) proteinuria	15.0%
					Grade 3/4 proteinuria	3.7%
Eric Van Cutsem et al. 2012 (19)	Prospective multinational, randomized, double-blind, parallel-arm, phase III study	1226	Metastatic Colorectal Cancer	Aflibercept plus chemotherapy	Proteinuria	All grades 62.2%
						Grade 3 7.5%
						Grade 4 0.3%
Ze-Feng Zhang et al. 2014 (17)	Meta -review	6882	solid tumors	VEGF-targeted	Proteinuria	All grade 18.7%
						Grade 3 or higher proteinuria 2.4%
Ze-Feng Zhang et al. 2014 (17)	Meta-review	535	Solid tumors	Axitinib	Proteinuria	All grade 20.2%
						High grade 4.6%
Dan Peng et al. 2023 (20)	Meta-review	510	Hepatocellular carcinoma	Apatinibversus sorafenib	Proteinuria	Incidence of proteinuria in the apatinib group was higher (RR = 2.58)
Lars Hofmann 2016 et al. (21)	Multi-center reporting	496	Metastatic melanoma	PD-1 monotherapy	IrAEs	27.8%
J.D.Wolchok.V et al. 2017 (22)	Prospective (double-blind trial)	1296	Advanced melanoma	CTLA-4 in combination with PD-1	IrAEs Grade≥3	96%
						∼59%
Jeffrey S. Weber, F et al. 2017 (23)	Pooled Analysis	576	Advanced melanoma	Nivolumab monotherapy	IrAEs Grade 3-4	71%
						10%
Cheng Xu et al. 2018 (24)	Meta-review	15370	Mainly lung cancer and melanoma	Both	IrAEs	54%-76%
Frank B.Cortazar et al. 2016 (25)	Comprehensive analysis	3695	Various	United	AKI	4.9%
Roman Shingarev et al. 2019 (26)	Review	11482	Various	CTLA-4 in combination with PD-1	AKI	2.1%
Harish Seethapathy et al. 2019 (27)	Retrospective observational cohort study	1016	Advanced malignancy	Both	AKI	17%
Sandhya Manohar et al. 2019 (28)	Meta-review	11482	varied from solid cancer to hematological malignancies	Pembrolizumab or nivolumab	AKI	2.2%
Sandhya Manohar 2020 (29)	Cohort study	1173	Predominantly metastatic melanoma	Monotherapy (CTLA-4 or PD-1)	AKI	2%
Harish Seethapathy et al. 2020 (30)	Retrospective cohort study	599	Advanced malignancy	PD-1	AKI	17%
Alejandro Meraz- Muñoz et al. 2020 (31)	Cohort study	309	Melanoma 262, lung 31, and genitourinary cancers 12.	Ipilimumab (70.9%) alone or in combination	AKI	16.5%
Busra Isik et al. 2021 (32)	Retrospective study	2143	The most common are lung cancer, melanoma, and renal cell carcinoma	Both	AKI	17%
Jonathan D. Sorah et al. 2021 (33)	Retrospective cohort study	1766	Predominantly non-small cell lung cancer (28%)	Monotherapy	AKI	7%
Maen Abdelrahim et al. 2021 (34)	10-year single-institution analysis	1664	Melanoma	Both	AKI	3.33%-3.49%
Clara Garcıá-Carro et al. 2022 (35)	Single-centre retrospective study	759	Mainly lung cancer	56% were receiving PD-1	AKI	15.5%
Megan L Baker et al. 2022 (36)	Observational cohort study	2207	Mainly lung cancer and melanoma	Any one of six ICIs	AKI	25%
Mehmet Kanbay et al. 2022 (37)	Review	3767	Lung cancer and melanoma predominate	Both	AKI	0-76%
Fei Liu et al. 2023 (38)	Meta	51141	Fifteen different tumor types	Both	AKI	1.4%
R Hájek et al. 2017 (39)	Randomized phase III study	315	Relapsed and refractory multiple myeloma	Carfilzomib	ARF	Carfilzomib, 8%
Paul G. Richardson et al. 2014 (40)	Randomized phase 2 study	221	Relapsed and refractory multiple myeloma	Pomalidomide	Renal failure and increased blood creatinine	The exact value is unknown
Harish Seethapathy et al. 2022 (41)	Retrospective cohort study	199	Melanoma	Dabrafenib, paired with trametinib	AKI	21%

AKI, Acute Kidney Injury; NSCLC, Non-small-cell lung cancer; mRCC, Metastatic renal cell carcinoma; PD-1, programmed death 1; CTLA-4, cytotoxic T lymphocyte-associated antigen 4; IrAE,Immune-related Adverse Effects; ARF,Acute Renal Failure.

**Table 2:** Mechanisms, pathological manifestations and clinical manifestations of renal injury caused by different antineoplastic drugs.

Drugs		Mechanisms	Clinical manifestations	Histopathology
**Platinum-derived compounds**	Cisplatin	Toxicity to tubular epithelial cells, vasoconstriction and pro-inflammatory effect of renal microvessels.	AKI, hypomagnesemia, hypokalemia, sodium depletion, fanconi-like syndrome and anemia.	Apoptosis and necrosis of the S3 segment of the proximal tubule, distal curved tubule, and collecting duct.
	Carboplatin			
	Ocaliplatin			
**Antimetabolites**	Methotrexate	Direct tubular injury with necrosis; decreased glomerular capillary perfusion; Affects folic acid metabolizing enzymes, impairs cellular RNA/DNA synthesis.	AKI, SIADH, hypertension, microangiopathic hemolytic anemia and ischemic skin lesions.	ATI,DITMA.
	Chlopharabine			
	Pemetrexed			
	Gemcitabine			
	Pentostatin			
**Anti-angiogenesis drugs (VEGF pathway inhibitors and TKI)**	Bevacizumab	Endothelial cell dysfunction and dysregulation of podocyte.	Hypertension, proteinuria and AKI.	Focal segmental glomerulosclerosis (FSGS), glomerular endothelial hyperplasia, cryoglobulinemia nephritis, nonspecific immune complex nephritis and acute interstitial nephritis.
	Sunitinib			
	Sorafenib			
**Immune checkpoint inhibitors (PD-1, PD-L1, CTLA-4)**	Ipilimumab	It is not clear. There may be multiple mechanisms of cell-mediated immunity and/or underlying autoimmunity.	Nephrotic syndrome, AKI, TMA, electrolyte disturbances.	Acute granulomatous interstitial nephritis and lupus membranous nephropathy.
	Pembrolizumab			
	Nivolumab			
**Alkylating agents**	Cyclophosphamide	Acrylic aldehyde is toxic to the bladder, and chloroacetaldehyde induces glutathione depletion and lipid peroxidation, damaging kidney tissue (7, 9, 44).	Bladder toxicity; proximal tubular acute tubular dysfunction; Fanconi syndrome; SIADH (7, 9).	Tubular cell damage or necrosis, mitochondrial swelling, and malformations (44).
	ifosphamide			
**Antitumor antibiotics**	Mitomycin C	Direct damage to the renal endothelium.	Hypertension; Urine sedimentation; Progressive renal failure.	The most common form is TMA.
**Immunomodulatory drugs**	Thalidomide	It is not clear.	Hypercalcemia; Decreased GFR; Nephrolithiasis.	Cases of acute interstitial nephritis and crystalline nephropathy have been reported.
	Lenalidomide			
	Pomalidomide			
**Anti-microtubular agents**	Vincristine	Affects the synthesis of genetic material such as DNA/RNA.	SIADH.	Kidney biopsy cases are lacking.
	Vinblastine			
	Vinorelbine			
**EGFR pathway inhibitors**	Cituximab	The mechanism of AKI is unknown; Inhibits the distal convoluted tubular EGFR signaling pathway and regulates epithelial transport of magnesium.	AKI, hypokalemia, hypocalcemia, and hypomagnesemia (7, 44).	Kidney biopsy cases are lacking.
	Panitumab			
	Nesitumab			
	Matuzumab			
**HER-2 inhibitors**	Trastuzumab	Unclear.	Proteinuria; AKI; Decreased GFR; Electrolyte disturbance; Hypertension.	Unclear.
	Pertuzumab			
**BCL-2 inhibitors**	Venetoclax	Tumor lysis syndrome.	AKI, hyperuricemia and hyperphosphatemia.	Kidney biopsy cases are lacking.
	Obituzumab			
	Ofatumumab			
**ALK inhibitors**	Crizotinib	Unclear.	Decreased GFR; Electrolyte disturbance (7, 45, 46).	Kidney biopsy cases are lacking.
**BRAF inhibitors**	Vimorafenib	Unclear.	Allergic interstitial diseases, acute tubular necrosis, proximal tubular injury (Fanconi syndrome), non-nephrotic proteinuria, acute/subacute GFR reduction of 20-40%, electrolyte disturbances.	Kidney biopsy cases are lacking.
	Dabrafenib			
**Cytokines**	IFN-a	The direct effect on podocytes may be promoted through receptor binding and activation: (1) weakening the proliferation and metabolism of podocytes; (2) Increase the oxidation capacity of podocytes and increase the expression of MHC class II antigens (44, 47).	Microvariable disease (MCD), FSGS and thrombotic microangiopathy (TMA).	Kidney biopsy cases are lacking.
	IL-2	Their effects are regulated by activating T cells, regulating receptor binding on T cells, memory T cells, and natural killer cells (7, 47).	Capillary leak syndrome; Cytokine-mediated inflammatory kidney injury.	Kidney biopsy cases are lacking.
	CAR-T	Cytokines mediate vasodilation and capillary leakage, causing prerenal injury, and CRS-associated hyperthermia and nausea and vomiting induce intravascular hypovolemia; Acute cardiomyopathy caused by CRS promotes hypotension and further aggravates renal hypoperfusion (48–51).	Electrolyte imbalance; Cytokine release syndrome; CRS-associated hyperthermia and nausea and vomiting.	Kidney biopsy cases are lacking.

AKI, Acute kidney injury; SIADH, Syndrome of inappropriate secretion of antidiuretic hormone; AIN, Acute interstitial nephritis; TMA, Thrombotic microangiopathy; DITMA, Drug-induced thrombotic microangiopathy; FSGS, Focal segmental glomerulosclerosis; GFR, Glomerular filtration rate; MCD, Microvariable disease; CRS, Cytokine release syndrome; IFN-a, Interferon-a; IL-2, Interleukin-2; CAR-T, Chimeric antigen receptor T-Cell immunotherapy.

### Treatments

Appropriate fluid therapy, correction of electrolyte abnormalities, prompt diuresis, and monitoring of renal function are particularly important to prevent platinum-based renal injury (53).There is currently no effective way to reverse AKI or tubular dysfunction, and treatment of renal toxic manifestations is primarily supportive with an aim to relieve symptoms. Advanced AKI exhibits uremia, metabolic derangement, and hypervolemia and requires dialysis, but dialysis is not effective in removing cisplatin. Intravenous saline or oral sodium chloride to correct hypovolemia and symptomatic hypomagnesium due to table salt, and intravenous/oral magnesium are necessary. Fanconi syndrome is notoriously difficult to treat (7, 9, 44).

## Antimetabolites

### Mechanisms and clinical manifestations

Methotrexate is widely used to treat malignancies such as advanced lymphoma, and its high-dose therapy (1-12 g/m^2^) is known to have an effect on renal function, with nephrotoxicity occurring during long-term therapy at conventional dose (54, 55). Dihydrofolate reductase inhibitors and their metabolites are filtered by the glomeruli and secreted from the proximal tubule to the cleft, and methotrexate favors crystal precipitation in the tubular lumen in a setting of slow urine flow rate and low urine pH, resulting in AKI due to direct tubular injury and necrosis (56). Methotrexate may also lead to a transient decrease in glomerular filtration rate due to a decrease in pressure because of contraction of glomerular capillary surface area and glomerular capillary perfusion, and afferent arteriolar or mesangial contraction. It can even cause syndrome of inappropriate secretion of antidiuretic hormone(SIADH) (7, 45).

Pemetrexed, a derivative of methotrexate excreted by the kidneys (70 to 90 percent within 24 hours, with a half-life of 3.5 hours), inhibits enzymes involved in purine/pyrimidine metabolism by affecting folate-metabolizing enzymes, impairs cellular RNA/DNA synthesis, and is approved for the treatment of advanced non-small cell lung cancer and pleural mesothelioma (44, 57) (58). Clofabine is also a purine nucleoside analog approved for the treatment of refractory childhood acute lymphoblastic leukemia and adult acute myeloid leukemia. It is possible that the inhibition of DNA synthesis, ribonucleotide reductase, and mitochondrial repair processes by clofarabine leads to ATI, while the ultimate understanding of mechanisms still requires renal biopsy. The nucleoside pyrimidine analogue, gemcitabine, is effective in the treatment of certain malignancies, mainly including lung, pancreatic, bladder, and breast cancers. Unfortunately, as with other chemotherapy drugs, many case reports and case series have documented AKI for gemcitabine, primarily TMA, which leads to drug-induced and dose-dependent thrombotic microangiopathy (DITMA) through immuno- and toxicity-mediated mechanisms.

In addition, hypertension, microangiopathic hemolytic anemia, and ischemic skin lesions may occur. The incidence and cases of renal adverse reactions caused by the antimetabolites above are detailed in **Table 1**.

### Treatments

Treatment involves the addition of folinic acid salvage to methotrexate to reduce nonmalignant cell damage and cleavage of methotrexate into noncytotoxic metabolites using glucosidase. High-throughput hemodialysis well clears plasma methotrexate (76 percent), but plasma concentrations may rebound after dialysis, and it can be used for severe acute kidney injury and may be unnecessary with the spread of glucosidase (44). In the case of immune-mediated DITMA, the best solution is to avoid the substance in question for life (7, 44).

## Anti-angiogenic drugs (VEGF pathway inhibitors and TKI)

### Mechanisms and clinical manifestations

**Figure 2:**
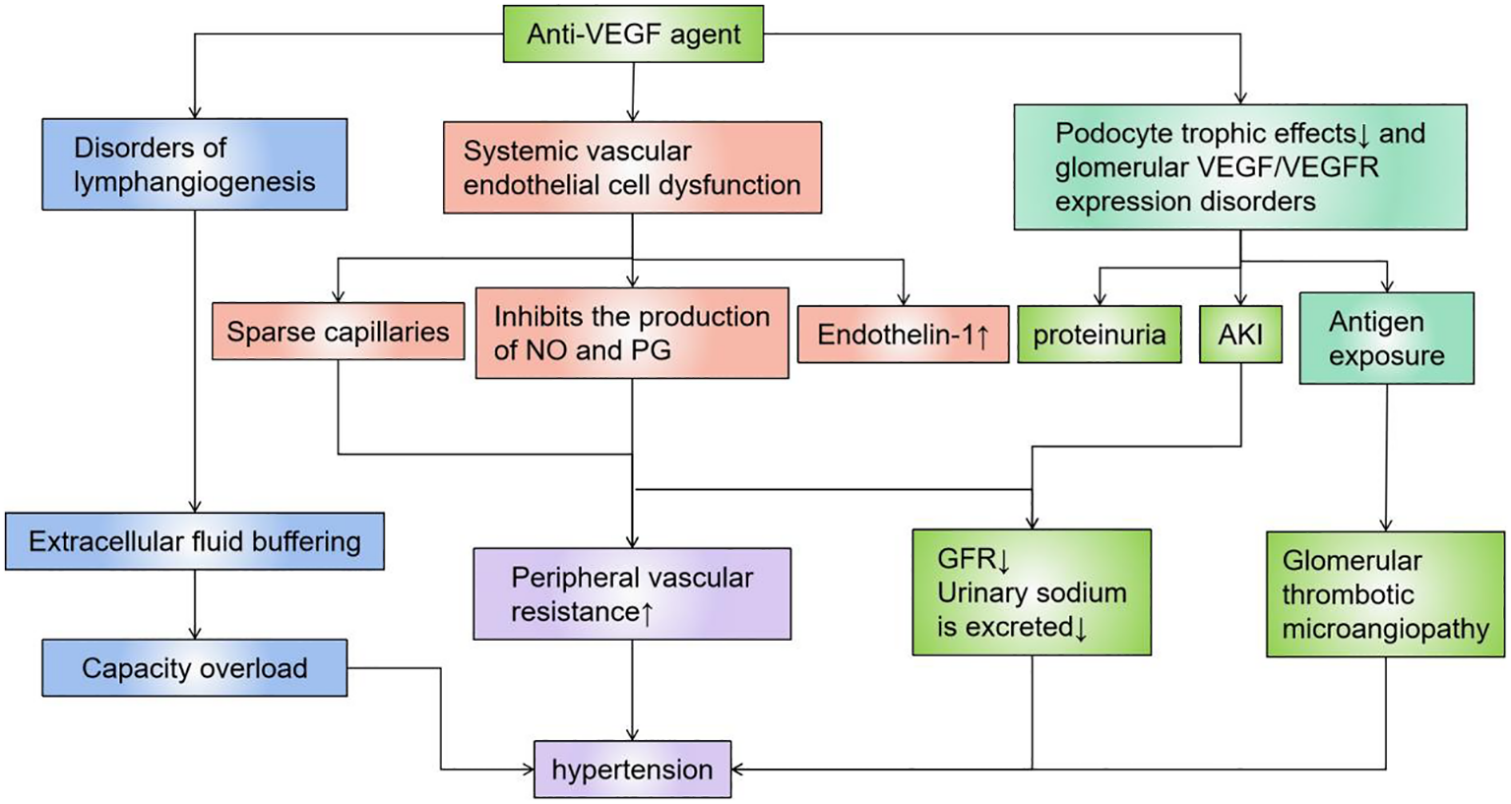
Mechanisms of action of anti-VEGF agents. VEGF is produced by renal visceral epithelial cells and binds to VEGF receptors located on the glomerular endothelium, mesangium, and periductal capillaries, and local VEGF synthesis is responsible for maintaining normal glomerular function and the integrity of the glomerular basement membrane, including damage repair and cell renewal. Anti-VEGF agents directly cause AKI by causing dysfunction such as vascular endothelium/lymphatic vessels, or podocytes, or indirectly cause kidney damage and hypertension by destroying blood vessels of the filtration system. VEGF, Vascular Endothelial Growth Factor.

The premise of this type of targeted drugs is that tumor growth is highly dependent on local pathological angiogenesis induced by vascular endothelial growth factor (VEGF), which promotes tumor development by regulating vascular permeability, endothelial cell migration, proliferation, and survival (44). VEGF is produced by renal visceral epithelial cells and binds to VEGF receptors located on the glomerular endothelium, mesangium, and periductal capillaries, while local VEGF synthesis is responsible for maintaining normal glomerular function and the integrity of the glomerular basement membrane, including damage repair and cell renewal (**Figure 2**). The most important and dose-dependent renal effects of antiangiogenic therapy are hypertension and kidney-specific impairment, including proteinuria and AKI (57, 59–62). However, the effects of antiangiogenic drugs are predominantly histopathologically manifested as thrombotic microangiopathy (**Figure 2**). Other lesions described on renal biopsy include focal segmental glomerulosclerosis (FSGS), parathyroid proliferative nephritis, glomerular endothelial hyperplasia, cryoglobulinaemic nephritis, nonspecific immune complex nephritis, and acute interstitial nephritis (63–65). These drugs are a logically targeted therapy that complements other tumor-targeted therapies. Similar renal TMA-like results can be seen due to the anti-VEGF effects ofTyrosine Kinase Inhibitors(TKIs) (66), and sunitinib or sorafenib can also cause acute and chronic interstitial nephritis, as well as chronic interstitial and endothelial damage, leading to CKD. Multitarget TKIs (eg, imani) and novel TKIs (eg, dasatinib and nilotinib) are associated with renal functional insufficiency and subsequent CKD (7, 9, 67). In animal experiments, the histopathology of the kidney of mice injected with a single dose of anti-VEGF antibody has shown glomerular endothelial cell swelling, vacuolization, detachment, and the rupture of the epithelial septum, while immunohistochemistry has also demonstrated that renin is down-regulated, and that the nephron could be partially restored with recombinant VEGF (68). There are also numerous reports of kidney injury associated with these drugs (**Table 1**).

### Treatments

Therefore, exacerbations in hypertensive patients on such drugs often predict a stronger response to antineoplastic therapy (69), which in turn motivates clinicians to continue antiangiogenic therapy while using antihypertensive drugs to control blood pressure rather than discontinuing antiangiogenic therapy.

## Immune checkpoint inhibitors (PD-1, PD-L1, CTLA-4)

### Mechanisms and clinical manifestations

**Figure 3:**
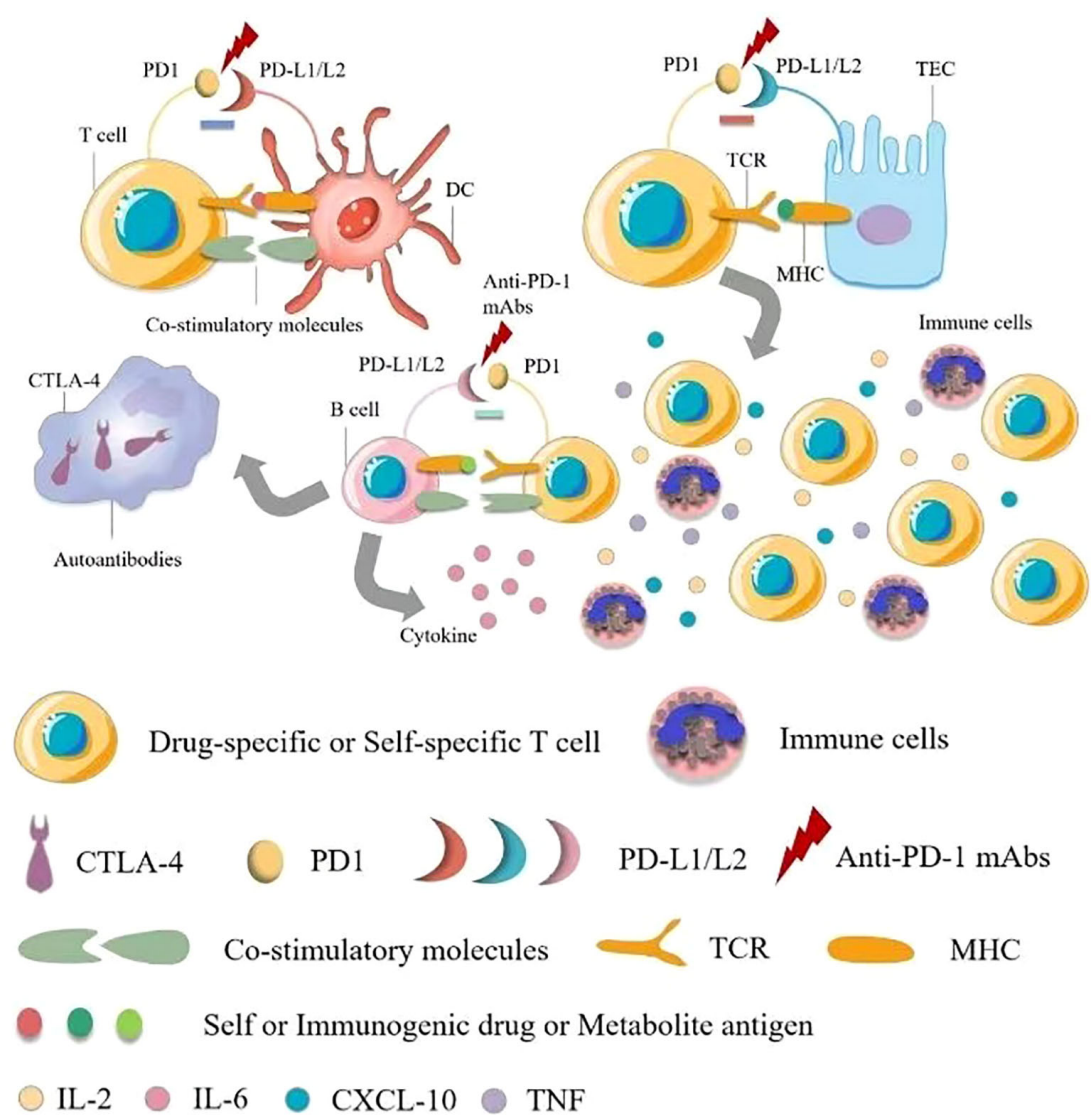
Pathophysiology of immune checkpoint inhibitor-associated acute kidney injury. There are multiple mechanisms of immune checkpoint inhibitor (ICI)-associated development of acute kidney injury (AKI). The first mechanism is loss of tolerance to autoantigens. Normally, effector-specific T cells are silenced by PD-1 signaling, which serves as a checkpoint for inhibiting T cell responses, and after using ICIs, T cells activate and cells are damaged. The second possible mechanism is through reactivation of drug-specific effector T cells. Proton pump inhibitors, antibiotics and other drugs can bind to tubular antigens and directly or indirectly trigger an immune response as haptens, which are trapped in the renal parenchyma, and reactive T cells can be dormant and activated when using ICIs. At the same time, serum cytokines such as CXCL10, TNF, and IL-6 levels are elevated, and administration may also lead to the production of autoantibodies against autoantigens presented by tubular epithelial cells, mesangial cells, or podocytes, resulting in an inflammatory environment. TCR, T cell receptor; TEC, tubular epithelial cells.

Immune checkpoint inhibitors (ICIs) are complementary therapies of cancers and are considered to be the most innovative and promising drugs in cancer treatment, including programmed death 1(PD-1), programmed cell death-Ligand 1(PD-L1), and cytotoxic T lymphocyte-associated antigen 4 (CTLA-4) monoclonal antibodies against these suppressor receptors expressed by tumor cells, T cells, and other immune cells. Immune-related Adverse Effects (IrAE) affects all systems, most commonly in the gastrointestinal tracts, endocrine glands, skin, and liver, and less commonly in the central nervous, cardiovascular, pulmonary, musculoskeletal, and hematologic systems (29, 70, 71). Some researchers have reported that ICIs-induced AKI may be caused by a unique mechanism of action involved in immune system reprogramming, leading to loss of tolerance (25). Pembrolizumab has also been reported to cause renal insufficiency (21). Ipilimumab is approved for treating advanced melanoma, and in a retrospective study of rare adverse events caused by ipilimumab, as reported in two cases of acute renal failure in 120 exposed patients, renal biopsy was more useful in examining renal manifestations (72, 73). Nephrotic syndrome, acute tubular injury and other manifestations of acute tubular necrosis in AKI have also been reported. Renal biopsy shows acute granulomatous interstitial nephritis and lupus membranous nephropathy (74). In some reports, steroid therapy has been associated with improved renal function (73, 75), although the mechanisms by which ipilimumab causes kidney disease are not well understood. Dual mechanisms are possible, including cell-mediated immunity (76) and/or underlying autoimmune mechanisms (74) (**Figure 3**).

### Treatments

The basic treatment for IrAEs is steroid medications, prednisone (0.5-1 mg/kg/day) or methylprednisone (1-2mg/kg/day). Of course, hydrocortisone is allowed in exceptional cases. In case reports, treatment with these agents has resulted in partial or complete remission, but relapse has also occurred (53).

## Other drugs

### Mechanisms and clinical manifestations

**Figure 4:**
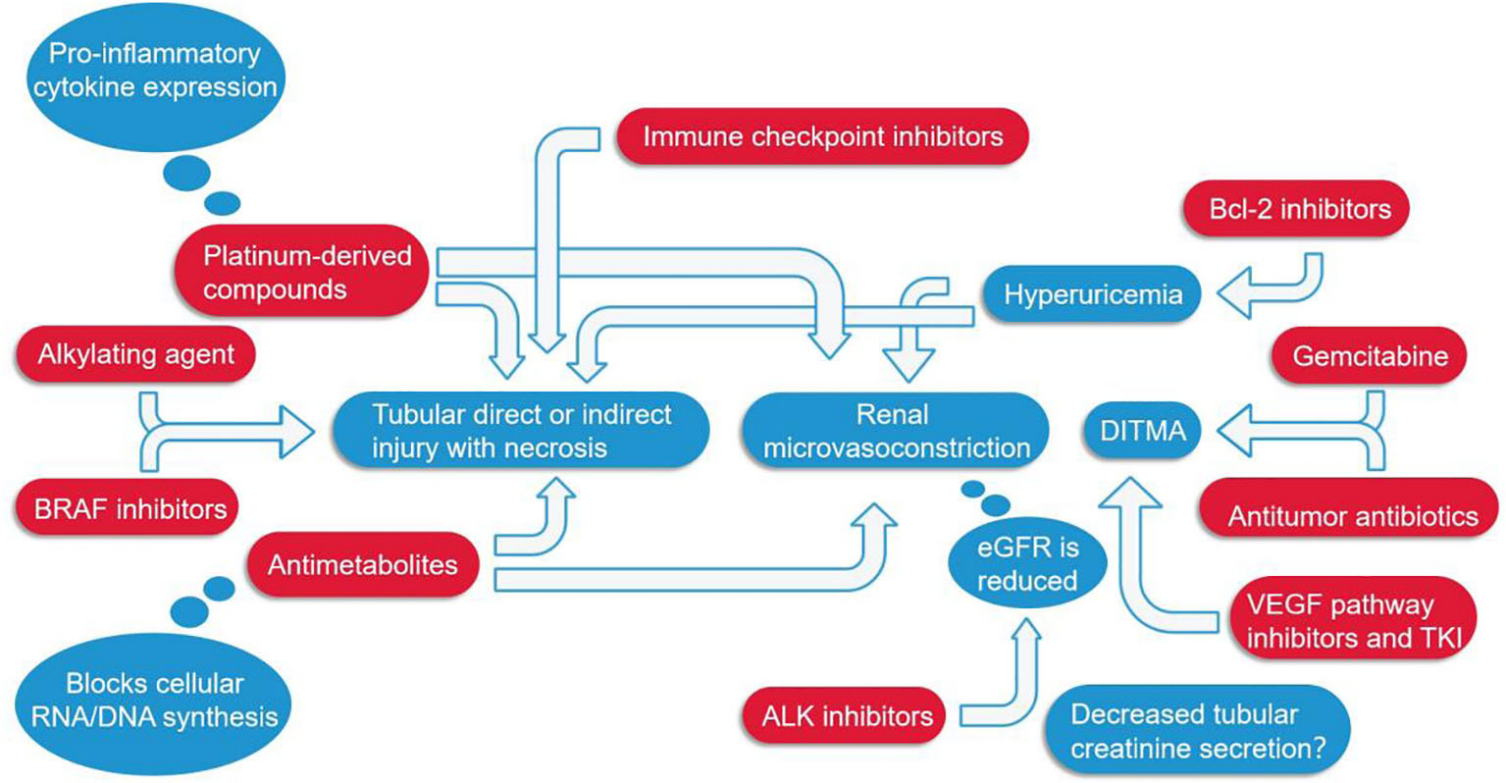
Several major classes of tumor drugs directly or indirectly cause renal function damage. Among them, ICIs can directly lead to renal tubular damage; Platinum-derived compounds cause tubular damage while also increasing the expression of pro-inflammatory cytokines; Antimetabolites mainly affect the synthesis of genetic material and can also cause constriction of renal microvessels; Anti-VEGF agents, antitumor antibiotics, and gemcitabine cause DITMA; Bcl-2 inhibitors increase uric acid, resulting in renal microvasoconstriction; Alkylating agents, BRAF inhibitors, ALK inhibitors, etc. also damage the renal tubules in different ways. DITMA, Drug-induced thrombotic microangiopathy.

Alkylating agents represented by cyclophosphamide and ifosfamide can generate cytotoxicity or nephrotoxicity and give rise to Fanconi syndrome through their metabolites. The antineoplastic antibiotic mitomycin C may cause nephrotoxicity through direct damage to the renal endothelium, which most likely occurs at least six months after treatment, with overall incidence depending on cumulative doses (7, 77). The immunomodulatory drug, thalidomide, and its derivatives are used in the treatment of multiple myeloma and have been reported to cause AKI (7, 45, 78–80). In addition to the programmed cell death protein 1 (PD-1) and its ligand (PD-L1), vascular endothelial growth factor (VEGR), and vascular endothelial growth factor receptor (VEGFR), the most commonly used cancer therapies target proteasomes, epidermal growth factor receptor (EGFR), human epidermal growth factor receptor-2 (HER2), HER2 dimer, v-Raf murine sarcoma virus oncogene homolog B (BRAF), anaplastic lymphoma kinase (ALK), mammalian targets of receptor activators (RANKL) and rapamycin (mTOR) of nuclear factor κβ ligands (7, 81). Monoclonal antibodies, such as cetuximab, have a 10-fold higher affinity for EGFR than natural ligands and can prevent EGF from binding to its receptor, making them an effective targeted therapy for these cancers. But, hypomagnesemia, hypokalemia, and hypocalcaemia may occur (7, 44). Trastuzumab is a recombinant humanized monoclonal antibody against the extracellular domain of HER2, while perstuzumab blocks the dimerization of HER2 required for cell activation and proliferation compared to trastuzumab, which can cause hypertension. Cardiorenal syndrome has been observed when combined with anthracycline antibiotics with cardiotoxicity, while reduced renal function in patients treated with trastuzumab means a higher risk of cardiotoxicity. Nephrotoxic effects also increase when used in combination with cisplatin-based drugs (7, 82). Inhibitors of B-cell lymphoma 2, most commonly used for hematologic tumors, are prone to tumor lysis syndrome with lysis of a large number of tumor cells, resulting in the release of a large amount of nucleic acid, phosphate and potassium into the systemic circulation, formation of nucleic acid metabolite uric acid, hyperuricemia, and uric acid precipitation in the renal tubules. In addition, hyperuricemia can lead to renal vasoconstriction, impaired autoregulation, decreased renal blood flow, and inflammation, resulting in nephrogenic AKI (7). The therapeutic small molecule inhibitor, crizotinib, is mesenchymal lymphoma kinase 1, which is used to treat non-small cell lung cancer. Clazotinib has been associated with a reduced eGFR and an increased risk of renal cyst development and progression (7, 45). Vemurafenib and dabrafenib are potent inhibitors of mutant BRAF kinase domains and are approved for the treatment of patients with advanced melanoma. Wanchoo et al. (83) summarized data of nephrotoxicity for these drugs, in which BRAF is expressed and localized in developing and mature glomerular podocytes in experimental animal models, while in a clinical setting, the putative mechanism of nephrotoxicity is tubular toxicity. Thus, renal biopsy has not yet been performed to elucidate the mechanisms behind the nephrotoxicity of BRAF inhibitors (7, 9, 45). Interferon and high dose IL-2 are the earliest immunotherapies clinically used to treat various malignant tumors, whereas new cancer immunotherapies have emerged in the past decade, such as the above-mentioned immune checkpoint inhibitors that activate quiescent T cells and impair the ability of tumors to turn off activated T cells in the tumor microenvironment. Chimeric antigen receptor (CAR) T cells are also emerging immunotherapies, which can be used to treat various malignant blood diseases and directly bind/destroy cancer cells, thereby overcoming the immune barrier utilized by cancer cells. Of course, nephrotoxicity is inevitable (**Figure 4**) (47).

### Treatments

First, adjusting the dose of cyclophosphamide and ifosfamide according to the degree of renal function can reduce nephrotoxicity. Mesna(antidote to acrolein) infusion and appropriate hydration can be used to prevent hemorrhagic cystitis. For complications of mitomycin use, the use of plasmapheresis and steroid therapy may suppress thrombotic microangiopathy without affecting the recovery or improvement of renal function (53).

It is important to emphasize that close monitoring, appropriate hydration, and dose reduction or even suspension of the use of the drug are preferred in these patients.

## The patient

The patient’s own baseline characteristics can increase the possibility of drug nephrotoxicity, such as relatively less muscle mass and total body water in women and the elderly, which may lead to drug overdose. These two groups of people have lower serum albumin concentrations, leading to decreased drug binding and thus increased drug free concentrations. In addition to this, other systemic and renal diseases may also increase the risk of nephrotoxicity of drugs. Diseases such as cirrhosis and nephrotic syndrome increase nephrotoxic effects through a variety of mechanisms. Examples include systemic effective volume reduction, renal hypoperfusion, hypoalbuminemia, and renal impairment not exhibiting clinical symptoms. For drugs cleared by the kidney, these complications increase nephrotoxicity through prerenal and renal mechanisms (84).

## Preclinical studies of drug-induced renal toxicity

### Kidney-on-a-chip technology

*In vitro* model that mimics kidney physiology and continuous blood flow conditions, can replicate the *in vivo* environment and predict the nephrotoxicity of certain drugs in the clinical setting (85). Proximal tubular cells cultured in this model show markers of various drug cytotoxicity and have been successfully applied to known nephrotoxins such as cisplatin (86).

### Cell- and biomarker-based assays

Biomarkers were added to the preclinical studies. Currently, routine biomarkers, such as serum creatinine and blood urea nitrogen, are the gold standard for assessing renal function and detecting kidney injury, including reversible increases in renal functional insufficiency and absolute increases in structural damage, although these two substances are not early biomarkers with abnormality measured only after nearly 50% GFR loss, limiting their use in clinics. In addition, their levels are affected by age, gender, food, fluid volume, muscle mass, medication, and other factors. Therefore, there is an urgent need for more sensitive biomarkers for early detection of AKI in the clinic. These potential novel biomarkers include neutrophil gelatinase-associated lipids (NGAL, also known as lipid 2), cluster proteins and kidney damage molecule-1 (KIM-1), polyheparin, albumin, total protein, β-2-microglobulin (B2M), cystinstatin C and trilobite 3 (TFF3), metalloproteinase tissue inhibitor 2 (TIMP2), insulin-like growth factor binding protein 7 (IGFBP7), and N-acetyl-d-glucosidase (NAG)(**Table 3**). In fact, pro-inflammatory interleukins, such as IL-6, IL-8, and IL-18, are also considered potential novel biomarkers for the detection of nephrotoxic kidney injury (7, 8, 45).

**Table 3:** Strengths and weaknesses of noninvasive markers of kidney injury.

Markers	Clinical applications	Deficiency
Serum creatinine and blood urea nitrogen	Reversible increase in functional kidney injury and absolute increase in structural damage.	Late biomarkers, abnormalities can only be measured after nearly 50% GFR loss.
NGAL	Urinary NGAL protein increases prior to elevated SCr, possibly as a molecule that distinguishes clinical AKI from subclinical or non-AKI patients (87).	Serum NGAL concentrations are weak for cisplatin-induced AKI detection (8).
Albumin	Patients with glomerular or tubular injury (88).	The accuracy is not strong.
β-2-M	Detection of β-2-M in urine indicates impaired tubular function.	Underlying malignancy also systematically increases β2-M and is not an accurate predictor of clinical and subclinical AKI.
NAG	Present in the proximal and distal tubules of rat renal tubules, which are generally not filterable, their presence in urine largely reflects tubular destruction.	There is no clear relationship between NAG changes and clinical diagnosis of AKI [105,107], and the utility of NAG for predicting cisplatin secondary clinical AKI is limited.
KIM-1	When cisplatin-induced AKI is clinically detectable, urine KIM-1 concentrations show a time-dependent increase, with peaks occurring earlier than SCr. The predictive value is strong.	The clinical relevance of KIM-1 alterations in subclinical AKI is not fully understood (89).
Cystatin C	Serum cystine C concentration is an endogenous marker of GFR, and the presence of cystine C in urine also indicates tubular damage.	Glucocorticoids, hyperthyroidism, and disorders affect serum concentrations, cystinstatin C may not be an ideal biomarker for cisplatin AKI (90–92).
CBP	Two conflicting studies observed an increase or decrease in CBP in the urine of rats treated with cisplatin, as well as an increase in calcium-binding protein in serum (93).	The mechanism of action of CBP in kidney injury is unclear.
IGFBP7 and metalloproteinase tissue inhibitor-2	IGFBP7 and metalloproteinase tissue inhibitor-2 are cell cycle inhibitors released into urine during kidney injury (94, 95).	More research is needed to further validate the efficacy of cisplatin therapy in patients in this trial and to find the best time for clinical application in patients at risk of AKI.
Clusterin	Anti-apoptotic properties are upregulated in renal injury (96, 97).	Not yet mentioned.
Urine protein MCP-1	Increased in the proximal tubule and urine of rats following cisplatin injury (98).	Not yet mentioned.
TFF3	Only one study quantified urinary TFF3 levels in patients after cisplatin treatment, in which an increase to a twofold increase to baseline was observed in patients not diagnosed with clinical AKI by traditional methods at day 10 (97).	Further studies are needed to understand why human TFF3 protein concentrations increase and rodents decrease after cisplatin exposure (89).
GST	Moderate elevation of GST-pi in the urine is a hallmark of distal tubular injury in cisplatin therapy without clinical AKI (97, 99).	Not yet mentioned.
FABP1	After cisplatin treatment, mouse FABP1 expressing human FABP1 protein was shed into urine, and the content was positively correlated with the degree of damage.	Quantitative studies of FABP1 in the clinical setting of cisplatin-induced AKI remain lacking (100, 101).
Osteopontine protein	Osteopontin is expected to become a biomarker.	Only one study evaluated the role of osteopontin in cisplatin-treated patients without clinical AKI, and no significant changes were found that warrant further study (102–106).
RBP	It is used as a diagnostic tool for proximal tubular lesions and renal interstitial disorders such as AIN, and can be used to guide treatment when renal biopsy is contraindicated (32, 107).	Not yet mentioned.
Urine TNF-α and IL-9	Distinguishes AIN from other causes of acute kidney disease (AKD) (107, 108).	Not yet mentioned.
FDG PET-ct	Several case reports of clinical diagnosis of ICPI-AIN on PET scan showed increased uptake of 18f-FDG in the renal cortex.	The reliability of this noninvasive and nonnephrotoxic approach still requires extensive research (107, 109).

NGAL, Neutropil gelatinase-associated lipocalin; GFR, Glomerular filtration rate; AKI, Acute kidney injury; Scr, Serum creatinine; β -2-M, β -2-Microglobulin; NAG, N-Acetyl-β -d glucosamine; KIM-1, Kidney injury molecule 1; CBP, Calcium-binding protein; IGFBP7, Insulin-like growth factor-binding protein-7; MCP-1, monocyte chemotaxis peptide-1; TFF-3, Recombinant human trefoil factor-3; GST, Glutathione transportase; FABP1, Fatty acid-binding protein 1; RBP, Retinol-binding protein; AIN, Acute interstitial nephritis; AKD, Acute kidney disease; FDG, fluorodeoxyglucose.

Kidney-on-a-chip technology, combined with new biomarkers, may give clinicians a better understanding of whether drugs are nephrotoxic. If so, the location of the injury and the underlying mechanism of kidney damage can be further understood.

## Conclusions

Oncological nephrology is a rapidly advancing field that demands close collaboration between oncologists and nephrologists. While the use of conventional and targeted therapies has improved patient outcomes, there have been reported complications due to the nephrotoxic effects of these anti-cancer drugs. Therefore, it is essential to gain a comprehensive understanding of the potential adverse effects associated with these treatments, enabling early detection and prevention of further complications. In this regard, it is crucial to emphasize the importance of integrating “omics” strategies, including the exploration of non-invasive biomarkers, such as urine-based markers, that can accurately reflect the severity of kidney injury and assist in risk stratification and prognostic assessment.

Future studies can appropriately focus on the following aspects: 1) the effects of potential renal dysfunction or complications of anti-tumor drugs in cancer patients. This allows a more comprehensive understanding of the risk of kidney injury in different patient populations. 2) Potential novel anti-tumor drugs that can reduce renal toxicity. 3)Further develop and validate biomarkers with high sensitivity and specificity.

## Author contributions

JT: Conceptualization, Data curation, Investigation, Methodology, Validation, Writing – original draft. NY: Conceptualization, Data curation, Investigation, Methodology, Validation, Writing – original draft. SP: Conceptualization, Data curation, Methodology, Supervision, Validation, Writing – original draft. PR: Conceptualization, Data curation, Investigation, Supervision, Writing – original draft. MC: Conceptualization, Data curation, Supervision, Writing – review & editing. JJ: Data curation, Funding acquisition, Supervision, Validation, Writing – review & editing. QH: Funding acquisition, Supervision, Validation, Writing – review & editing. YZ: Conceptualization, Data curation, Funding acquisition, Methodology, Resources, Supervision, Validation, Writing – review & editing.
